# Plant-Derived Tragacanth Gum Coating Ameliorates Chilling Injury and Maintains Eating Quality by Downregulating Oxidative Stress in Loquat Cultivars

**DOI:** 10.3390/foods15142437

**Published:** 2026-07-09

**Authors:** Mian Muhammad Ahmed, Muqaddas, Muhammad Asim, Saqib Ayyub, Muhammad Atiq Ashraf, Ahmad Sattar Khan, Zhang Na

**Affiliations:** 1 College of Life Science and Technology, Tarim University, Alar 843300, China; mian.ahmed1@outlook.com; 2 Postharvest Research and Training Centre, Institute of Horticultural Sciences, University of Agriculture, Faisalabad 38040, Punjab, Pakistan; asim@webmail.hzau.edu.cn (M.A.); saqibayyub218@gmail.com (S.A.); atiqashraf25@gmail.com (M.A.A.); 3 College of Food Science and Engineering, Tarim University, Alar 843300, China; muqaddas031@gmail.com; 4 National Key Laboratory for Germplasm Innovation and Utilization of Horticultural Crops, College of Horticulture and Forestry Sciences, Huazhong Agricultural University, Wuhan 430070, China

**Keywords:** antioxidant activity, oxidative stress, edible coating, lipid peroxidation, phytochemical accumulation, shelf period

## Abstract

Loquat is a highly perishable, non-climacteric fruit that is prone to chilling injury, decay, and eating quality deterioration during cold storage. This study evaluates the effectiveness of tragacanth gum (TG) as a plant-based edible coating to mitigate postharvest chilling injury (CI) and preserve the eating quality of loquat fruit. Two commercial cultivars, ‘Sufaid’ and ‘Surkh’, were coated with 1% TG and stored at 4 ± 1 °C with 90 ± 5% relative humidity for 20 days, followed by 2 days at 20 °C ± 1 °C (specific focus on retail handling). TG-coated fruits exhibited significantly reduced weight loss and lower decay compared to controls. TG application alleviated CI symptoms and reduced respiration rates. TG-treated fruits maintained higher levels of titratable acidity, total soluble solids, and ascorbic acid. TG application also elevated phytochemical levels and antioxidant potential, reinforcing the fruit’s biochemical defenses against postharvest stress. Furthermore, TG treatment suppressed oxidative stress by lowering malondialdehyde and hydrogen peroxide levels and enhanced the activities of key antioxidant enzymes (SOD, CAT and APX). TG-coated fruits also showed improved taste, flavor, and consumer acceptability at shelf after storage. In conclusion, results indicate that application of TG coating represents a sustainable, eco-friendly strategy to alleviate CI and improve postharvest eating quality of commercial loquat cultivars, offering potential for broader adoption in the fresh fruit supply chain.

## 1. Introduction

Loquat (*Eriobotrya japonica* Lindl.) also known as Japanese plum or Chinese plum is a well-known pome fruit native to southeastern China. It is believed to have originated in China over 2000 years ago and has since been widely cultivated in other parts of Asia, including Japan, Korea, and Southeast Asia [1]. It is produced in China, Japan, Pakistan, Egypt, Greece, Morocco, Portugal, Italy, Turkey, Chile, Brazil and Spain. China is currently the world’s largest producer of loquat, cultivating approximately 170,000 ha and producing around one million tons annually [2]. In Pakistan, it is cultivated on a 1515 ha area with 10,482 tons produced annually [3]. Khyber Pakhtunkhwa (KPK) and Punjab province mainly grow two indigenous cultivars, “Sufaid” and “Surkh” in Pakistan. Due to its pleasant flavor and appealing yellow color, the loquat fruit has great economic and medical value [4]. In terms of nutrition, it has malic acid, sucrose, and trace amounts of citric, succinic, and tartaric acids. In addition, it has a high concentration of carotenoids, particularly provitamin A [5]. Because of non-climacteric nature, it has a relatively short shelf life and is vulnerable to deterioration due to quick senescence after harvest. It has greater postharvest losses due to poor management practices. It can be kept under low-temperature (0–5 °C) storage for 3 to 4 weeks depending on the cultivars and treatments that were used [6].

An economically significant postharvest issue known as chilling injury (CI) lowers the overall quality and marketability of many tropical and subtropical fruits and vegetables. Excess reactive oxygen species (ROS) can cause peroxidation and the breakdown of unsaturated fatty acids in membrane lipids, which can lead to the development of CI damage symptoms [7]. On the other hand, ROS scavengers function as antioxidants to reduce the severity of the chilling injury and enhance the degree of unsaturation of 18-carbon fatty acids in the polar lipids [8]. A variety of interconnected antioxidant enzymes, including superoxide dismutase (SOD), catalase (CAT), and ascorbate peroxidase (APX), regulate the metabolism of ROS. SOD effectively converts the superoxide anion radical (O_2_^−^) into H_2_O_2_, while APX and CAT mostly destroy H_2_O_2_ [9]. Previous research has demonstrated a positive correlation between the antioxidant enzyme activity and the harvested fruit’s tolerance to chilling injury [10,11,12]. These findings imply that increased antioxidant enzyme activity and decreased membrane lipid peroxidation may contribute to the ability of harvested fruit to withstand chilling injury.

Due to consumer demand for chemical-free food, plant-based antimicrobial coatings are widely used worldwide. Natural alternatives like cinnamon oil, gum arabic, lemon grass, and tragacanth gum (TG) are crucial for preventing fruit contamination, improving quality, and extending storage life [13]. Edible coatings act as a barrier to exchanging gases. These coatings reduce the moisture loss that reduces weight loss, respiration rate, and also inhibit microbial growth, promote color and delay changes in firmness [14]. TG is a plant-based acidic polysaccharide. *Astragalus gummifer* is among the most prevalent species that exude tragacanth gum [15]. A polysaccharide-based compound known as TG, which is biodegradable and biocompatible, falls into the ‘generally regarded as safe’ category when applied from 0.2% to 1.3% concentration. There have been reports on the application of TG on a variety of fruits and vegetables. The weight loss, decay, electrolyte leakage, formation of hydrogen peroxide, and malondialdehyde were all significantly reduced in apricot fruits coated with 1% TG [16]. The use of 1% TG has been found appropriate in conserving quality, maintaining fruit weight and reduced decay of cherry [17]. Application of 1.5% TG slowed the softening of mango fruit, thereby delaying ripening and extending postharvest shelf life [18]. However, the use of TG coatings on loquat fruit has not been reported yet.

Edible coatings of different types such as cactus pear mucilage [19], black cumin oil [20], cherry and apricot tree gums [21], propolis extract + cinnamon oil [22], gum arabic [5], and *Torreya grandis* essential oil [23] have already been reported on loquat fruit. A completely adequate edible coating type for application on loquat fruits has not yet been reported that maintains quality during cold storage followed by shelf period. Therefore, it is crucial to look for a suitable edible coating that may be utilized to prolong the shelf life of loquat fruits during cold storage. Currently, there is a lack of literature on postharvest treatment of TG for reducing losses and managing quality of loquat fruit during storage and shelf. This study aims to investigate the impact of TG application on storage life and quality, filling this knowledge gap to benefit researchers and the loquat industry.

## 2. Materials and Methods

### 2.1. Fruit Material

The study was conducted at Postharvest Research and Training Centre (PRTC), Institute of Horticultural Sciences (IHS), University of Agriculture Faisalabad (UAF), Pakistan. For the experiments, the fruit of both cultivars ‘Surkh and Sufaid’ was harvested from Haji Haneef Loquat Orchard (32°46′15.9″ N; 72°41′42.1″ E) Kallar Kahar, Punjab, Pakistan. Fruit graded for uniform size, color, free from any symptoms of diseases and insect pests and pre-cooled at 10 ± 1 °C was transported in a refrigerated vehicle to PRTC, IHS, UAF, Pakistan within 4 h. After cleaning the fruits with clean tap water, 0.1% NaOCl was used for 3 min to surface-sterilize them. Fruits were then spread out on a table and allowed to dry at room temperature before being utilized in the study.

### 2.2. Preparation of Tragacanth Gum and Treatment Application

The tragacanth gum powder (Sigma-Aldrich, St Louis, MO, USA) was dissolved in sterilized distilled water and extensively homogenized for about 2 h at 70 °C on a magnetic stirrer before being maintained at 4 °C for 24 h for through hydration [24]. For the purpose of plasticizing and surfactant, glycerol (1%) and Tween-20 (0.01%) were added. After that, the coating was mixed for 5 min in a blender (WF-8813, Westpoint, Faisalabad, Pakistan) and allowed to cool for 1 h. The loquat fruits were immersed in the coating solution for 3 min at room temperature, with the final concentration being 1%. During an initial experiment, which applied several concentrations (0.25%, 0.50%, and 1%) of gum, the employed concentration (1%) was optimized. Fruits of both Surkh and Sufaid were placed in cardboard boxes and stored at 4 ± 1 °C with 90 ± 5% of R.H. for 20 days. Fruits were stored under cold conditions, and physical, biochemical, phytochemical, sensory and enzymatic quality attributes were assessed at 5-day intervals; at each sampling point, fruits were removed from cold storage, held at room (ambient) temperature (20 °C ± 1 °C) for 2 days, and then data was recorded.

### 2.3. Assessment of Fruit Weight Loss, Firmness and Respiration Rate

The measurement of physiological weight loss (WL) in fruit was conducted by comparing the initial and final weights of each replication on the designated sampling days. Weight loss was expressed as a percentage (%). Fruit firmness was measured at two equatorial points after removing the peel. A digital fruit firmness tester (FR-5120, Lutron, Norfolk, VA, USA) equipped with a 6 mm plunger tip was used, and the firmness values were recorded in Newtons (N) [25,26]. Respiration rate was determined as CO_2_ production from the fruits. Two fruits were placed in a sealed 1 L airtight glass jar fitted with a rubber septum. After 1 h of incubation, 2 mL of headspace gas was withdrawn and injected into an infrared gas analyzer (Analyser Series 1450 Food Package Analyser; Servomex Ltd., Crowborough, East Sussex, UK). The CO_2_ peak area was compared against a 2 mL CO_2_ standard (8.2% in N_2_; BOC Gases, Perth, Australia), and the respiration rate was expressed as mmol CO_2_ kg^−1^ h^−1^ [25].

### 2.4. Evaluation of Chilling Injury, Decay Incidence and Marketability Index

Each fruit was checked visually for the symptoms of CI at the time of data collection. The level of chilling injury was examined by the symptoms of surface pitting, which were awarded a score. The scale that goes from 1 to 5 was used as explained by [27]. Decay index was evaluated by observing infected fruit from the total number of fruits and the reading was expressed as a score. Fruits exhibiting rotting symptoms were declared as decayed fruit. Decay incidence was expressed in score from 1 to 9 where 1 is the least decay and 9 is maximum decay [28]. The marketability index (MI) of each replication in experiment was determined by dividing the healthy fruits (decay, disease and injury free) with total number of fruits. The marketability index was expressed in %.

### 2.5. Determination of Total Soluble Solids, Titratable Acidity and Ascorbic Acid Content

To examine total soluble solids (TSS), the juice of each replication was used, and two readings were noted from each replication by using Digital Refractometer Model no. ATAGO, RX 5000, Tokyo, Japan. The mean value of each replication was calculated in °Brix. Titratable acidity (TA) was measured by using a technique given by Hortwitz [29]. To assess the TA, a 10 mL juice sample of loquat was taken in beaker and 20 mL of distilled water was added, after mixing 2 to 3 drops of indicator (Phenolphthalein) were added in a beaker and shaken well. The final solution was titrated against 0.1 N solution of NaOH with dropwise until a pink color appeared. The mL of NaOH was noticed and TA was measured in percentage by using the formula:

TA (%) = (Volume of NaOH used (mL) × 0.0067)/(Loquat fruit juice (mL))

The 2,6-dichlorophenol-indophenol technique was used to measure the ascorbic acid content (AsA) of loquat flesh. Based on the dye volume titration used, the ascorbic acid content was calculated and given in mg 100 g^−1^ FW [30].

### 2.6. Quantification of Total Phenolics, Carotenoids Content and Antioxidant Activity

The total phenolic content (TPC) concentration was determined using the Folin–Ciocalteau test. The absorbance was measured using a spectrophotometer (UV-1800, Shimadzu, Kyoto, Japan) at 765 nm. Total phenolic concentration was computed and a standard curve for gallic acid was created. Finally, mg GAE 100 g^−1^ FW was used to express the TPC [31]. The total carotenoid contents of the loquat fruit were assessed by the method as expressed by [16] with minor changes. The carotenoid contents of the fruit pulp were calculated as mg kg^−1^ FW. For this, 1 g frozen stored sample of loquat fruit pulp was ground using a mortar and pestle in 10 mL extraction mixture of n-hexane (C_6_H_14_) and acetone (C_3_H_6_O) (6:4). The mixture was adequately homogenized and incubated for automatic separation of supernatant and pellets. The supernatant was then shifted into UV-Vis cuvette and absorbance was noticed at 453, 505, 645 and 663 nm at the same time with the help of a spectrophotometer. Finally, the following formula was used to calculate carotenoid contents:

Carotenoids (mg kg^−1^ FW) = (0.452A_453_ − 0.304A_505_ − 1.22A_645_ − 0.216A_663_) × 1000

The total antioxidant activity of loquat fruit pulp during ripening was determined following the method described in [25,32] and expressed as µM Trolox 100 g^−1^ fresh weight (FW).

### 2.7. Measurement of Malondialdehyde, Hydrogen Peroxide and Electrolyte Leakage

The total malondialdehyde contents (MDA) of the loquat fruit were assessed by the method as stated by [26] and expressed in µmol kg^−1^ FW. Briefly, 1 g of fruit sample was homogenized in 10 mL of 10% (*w*/*v*) TCA and centrifuged at 12,000× *g* for 10 min. Subsequently, 1 mL of the supernatant was mixed with 1 mL of 0.6% 2-thiobarbituric acid (TBA) and heated in boiling water for 5 min, followed by cooling on ice. The mixture was then centrifuged at 10,000× *g* for 5 min, and the absorbance of the supernatant was measured using a spectrophotometer at 450, 532 nm and 600 nm. MDA level was determined using the following formula:

MDA contents (µmol kg^−1^ FW) = [6.45 × (A_532_ − A_600_) − 0.56 × A_450_]

Total hydrogen peroxide (H_2_O_2_) levels in loquat fruit were measured using the [33] method, and the results were given in µmol kg^−1^ FW. For this, a 1 g frozen fruit pulp sample was completely homogenized in 1 mL TCA (0.1%) in an ice bath and centrifuged at 12,000× *g* for 15 min. After that, 0.5 mL supernatant was combined with 0.5 mL of K_3_PO_4_ buffer (10 mM) and 1 mL of 1M KI reagent. Finally, the assay (200 µL) was subjected to the spectrophotometer, and the absorbance was examined at 390 nm. Ion leakage or electrolyte leakage (EL) of loquat fruit was measured with the help of a method expressed by [34] with some modifications. For this purpose, five equal small disk-like pieces of loquat fruit were removed with the help of a cork borer. In a glass beaker, 50 mL of distilled water was used to dip these pieces. The first reading of electrical conductivity (EC-1) was measured at (20 °C ± 1 °C) after 30 min with the help of a digital EC meter (CD-4301, Lutron Inst. CO., London, UK). Then the solution was boiled in a microwave oven. After cooling down, a second reading of (EC-2) was taken again. Finally, the ion leakage was measured with the help of the following formula and expressed in percentage:

EL (%age) = (EL − 1)/(EL − 2) × 100

### 2.8. Enzymatic Analysis (SOD, POD, CAT, and APX)

Enzyme extraction from loquat samples was carried out following the detailed protocol described by [16]. For this, 1 g of loquat flesh was thoroughly homogenized in 2 mL of phosphate buffer (pH 7.2). The homogenate was then centrifuged at 10,000× *g* for 10 min, and the resulting supernatant was used to measure the activities of antioxidant enzymes, including APX, CAT, peroxidase (POD) and SOD. The activities of POD, CAT, and SOD were determined according to the method of [35]. Specifically, POD activity was measured by tracking the formation of tetraguaiacol from guaiacol at 470 nm, while CAT activity was determined by monitoring the decomposition of hydrogen peroxide at 240 nm. SOD activity was analyzed using the nitro blue tetrazolium (NBT) method, recording absorbance at 560 nm. APX activity was assessed by monitoring the oxidation of L-ascorbate in the presence of hydrogen peroxide, measuring the absorbance at 290 nm [36]. All enzyme activities were expressed as U mg^−1^ protein, and total protein content was quantified [37].

### 2.9. Sensory Evaluation

Sensory analysis was performed according to a previously described method [6]. The assessment was conducted by a panel of 10 trained assessors. The taste, flavor and overall acceptability of loquat fruit were evaluated using a 9-point hedonic scale where 1 = extremely disliked, 5 = neither liked nor disliked and 9 = extremely liked. The evaluations were conducted under uniform laboratory conditions at 20 ± 1 °C. Each treatment was replicated three times for all sampling intervals. Coated and uncoated loquat fruits were served on disposable plates with randomly assigned two-digit codes and presented in a randomized blind sequence immediately after removal from storage. The panelists were instructed to cleanse their palates with crackers and water in between samples.

### 2.10. Data Analysis

Data for each cultivar (Sufaid and Surkh) were analyzed separately using a two-factor factorial CRD with treatment and storage period as factors. Each treatment was replicated three times, with 12 fruits per replicate. Data were analyzed using Statistix 8.1 software (Tallahassee, FL, USA), and graphs were created in Microsoft Excel (Microsoft 365). The effects of treatments, storage period, and their interaction were evaluated using two-way ANOVA, and differences between means were compared using the LSD test at *p* ≤ 0.05 [38]. Correlations between the various physiological, biochemical and quality parameters measured were calculated using the R programming language (R 4.5.0 (2025)) (R Core Team, Auckland, New Zealand) with use of the complexHeatmap package, which is designed to create clustered correlation heat maps and represent the relationships between the variables.

## 3. Results

### 3.1. Fruit Weight Loss, Firmness and Rate of Respiration

**Figure 1:**
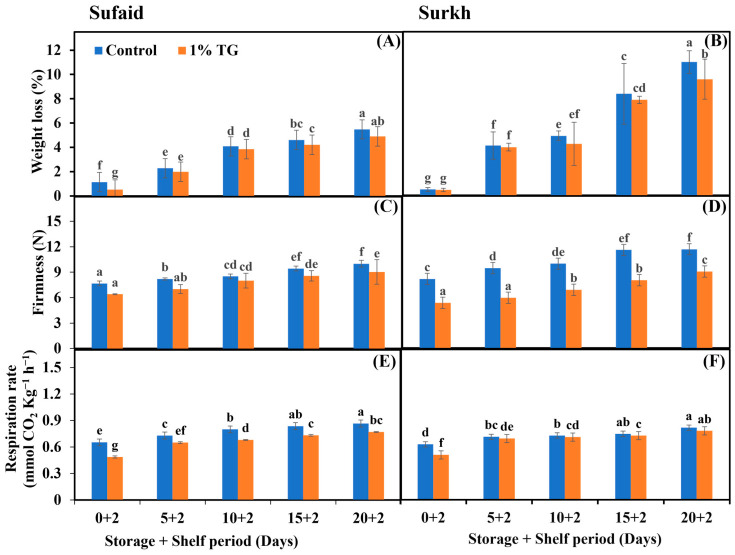
Effect of postharvest TG coating on changes in weight loss (**A**,**B**), firmness (**C**,**D**) and respiration rate (**E**,**F**) of loquat cvs. Sufaid and Surkh fruits kept at cold storage and 2-day shelf period. Different letters above bars indicate significant differences between treatments at *p* ≤ 0.05 (LSD test). Error bars represent ± SE; *n* = 36 (12 fruits × 3 replications).

Fruit weight loss increased progressively with advancing cold-storage interval when assessed after the standardized 2-day shelf period at (20 °C ± 1 °C) in both loquat cultivars (Sufaid and Surkh). In ‘Sufaid’, TG application reduced mass loss relative to the control across intervals, with the clearest separation observed after 20 days of cold storage plus 2 days of shelf period at (20 °C ± 1 °C), where TG-treated fruit showed lower weight loss (4.90%) than the control (5.48%) (Figure 1A). In ‘Surkh’, weight loss likewise rose with increasing storage interval, and TG-coated fruit consistently maintained lower weight loss than non-treated fruit, reaching 9.23% versus 10.60% after 20 days of cold storage plus 2 days of shelf period at 20 °C ± 1 °C (Figure 1B). Significant differences were observed at last assessment point defined as cold storage followed by 2 days of shelf period at 20 °C ± 1 °C, where control fruits exhibited higher weight loss compared to treated fruits. Fruit firmness increased progressively during storage after 2 days of shelf life at 20 °C ± 1 °C, with a more pronounced increase in ‘Surkh’ compared to ‘Sufaid’. In ‘Sufaid’, control fruit consistently exhibited higher firmness than TG-treated fruit at the later storage intervals, particularly after 20 days of cold storage plus 2 days of shelf period, where control fruit recorded 10.0 N compared with 9.03 N in TG-treated fruit (Figure 1C). Similarly, in ‘Surkh’, control fruit maintained higher firmness (11.62 N) than TG-treated fruit (9.00 N) after the same storage and shelf period duration (Figure 1D). Overall, fruit firmness increased with storage duration in both cultivars; however, TG treatment had a minimal or slightly reducing effect on textural firmness in both cultivars. Respiration rate increased with storage progression in both cultivars when measured after the fixed 2-day shelf period at (20 °C ± 1 °C) period following each cold-storage interval. In ‘Sufaid’, TG consistently suppressed respiration relative to the control at the later intervals; after 20 days of cold storage plus 2 days of shelf period at 20 °C ± 1 °C, TG-treated fruit showed a lower respiration rate (0.77 mmol CO_2_ kg^−1^ h^−1^) than control fruit (0.87 mmol CO_2_ kg^−1^ h^−1^) (Figure 1E). In ‘Surkh’, TG similarly reduced respiration at the final assessment point, with TG-treated fruit recording 0.78 mmol CO_2_ kg^−1^ h^−1^ compared with 0.81 mmol CO_2_ kg^−1^ h^−1^ in the control after 20 days of cold storage plus 2 days of shelf period at 20 °C ± 1 °C (Figure 1F). Thus, TG moderated respiratory activity under the defined evaluation condition of cold storage followed by 2 days of shelf period at 20 °C ± 1 °C.

### 3.2. Chilling Injury, Decay Index and Marketability Index

**Figure 2:**
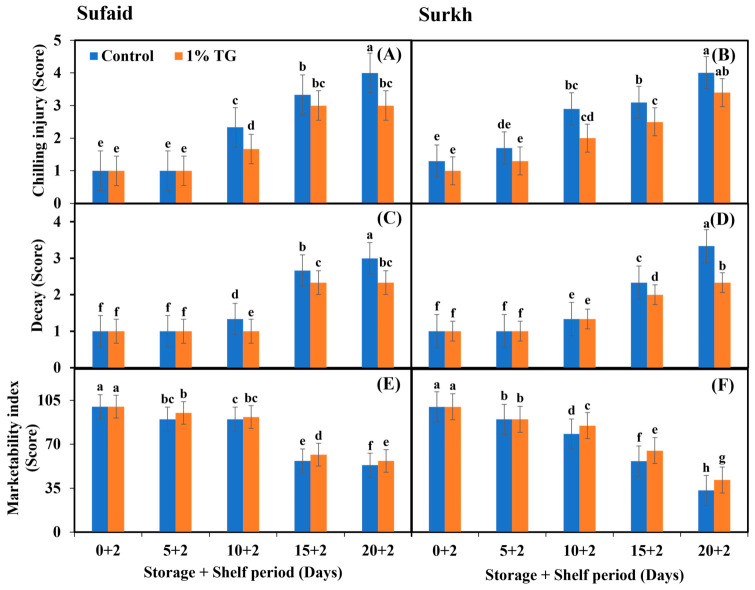
Effect of postharvest TG coating on changes in chilling injury (**A**,**B**), decay (**C**,**D**) and marketability index (**E**,**F**) of loquat cvs. Sufaid and Surkh fruits kept at cold storage and 2-day shelf period. Different letters above bars indicate significant differences between treatments at *p* ≤ 0.05 (LSD test). Error bars represent ± SE; n = 36 (12 fruits × 3 replications).

**Figure 3:**
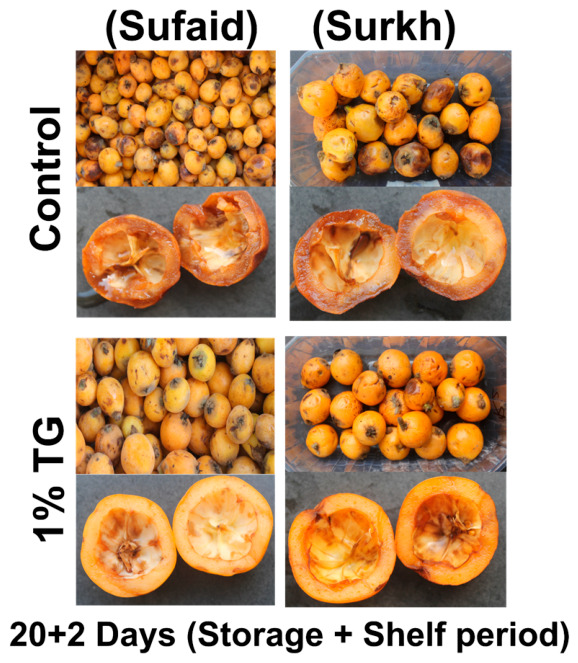
Visual representation (external and half-cut) of TG coated and control loquat fruits (cvs. Sufaid and Surkh) during 20 days of cold storage and 2 days shelf period at 20 °C ± 1 °C.

Chilling injury increased with advancing storage interval in both loquat cultivars (Sufaid and Surkh) when assessed after 2 days at 20 °C ± 1 °C following each cold-storage interval. In ‘Sufaid’, chilling injury differed significantly across storage intervals (*p* ≤ 0.05), and by 20 days of cold storage plus 2 days at 20 °C ± 1 °C, 1% TG-treated fruit showed a lower injury score (3.0) than the control (3.3) (Figure 2A). In ‘Surkh’, storage interval had a highly significant effect on chilling injury (*p* ≤ 0.01), and TG-coated fruit consistently exhibited lower injury; at 20 days of cold storage plus 2 days at 20 °C ± 1 °C, TG-treated fruit recorded an injury score of 3.4 compared with 4.0 in the control (Figure 2B). Fruit decay increased with advancing storage interval in both cultivars when evaluated after 2 days at 20 °C ± 1 °C following each cold-storage interval. In ‘Sufaid’, decay was significantly influenced by treatment, storage interval, and their interaction (*p* ≤ 0.05), with TG reducing decay at 20 days of cold storage plus 2 days at 20 °C ± 1 °C (2.3) compared with the control (3.0) (Figure 2C). In ‘Surkh’, decay increased significantly with storage interval (*p* ≤ 0.01), and TG-coated fruit maintained lower decay at the final assessment point, showing 2.3 versus 3.3 in control after 20 days of cold storage plus 2 days at 20 °C ± 1 °C (Figure 2D). Figure 3 shows external and half-cut views of TG-coated and control loquat fruits (cvs. Sufaid and Surkh) during storage and shelf period, highlighting chilling injury and decay development.

Marketability index declined progressively with advancing storage interval in both loquat cultivars when assessed after 2 days at 20 °C ± 1 °C following each cold-storage interval. In ‘Sufaid’, storage interval and treatment significantly affected marketability (*p* ≤ 0.05), while their interaction was non-significant; at 20 days of cold storage plus 2 days at 20 °C ± 1 °C, 1% TG produced the highest marketability score (56.7) compared with the control (53.3) (Figure 2E). In ‘Surkh’, storage interval and treatment also significantly influenced marketability (*p* ≤ 0.05), with treated fruit retaining higher marketability at the final assessment point (41.7) than the control (33.3) after 20 days of cold storage plus 2 days at 20 °C ± 1 °C (Figure 2F).

### 3.3. TSS, TA and Ascorbic Acid Content

**Figure 4:**
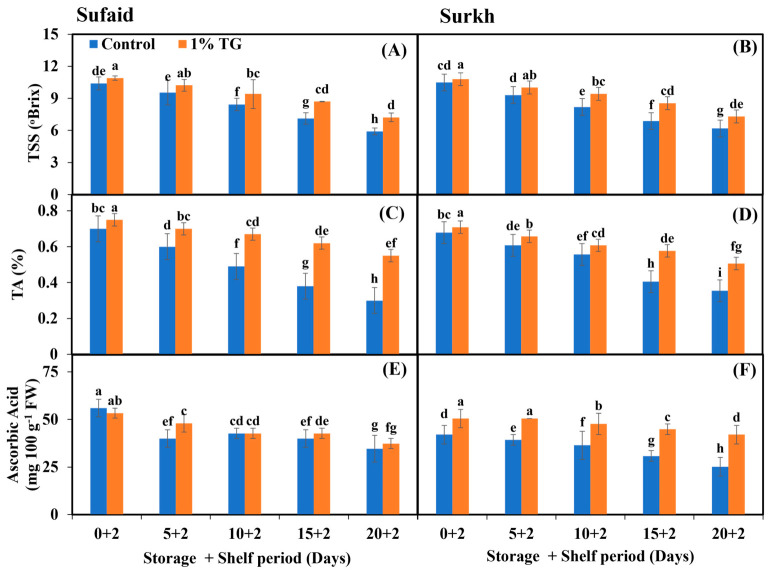
Effect of postharvest TG coating on changes in TSS (**A**,**B**), TA (**C**,**D**) and ascorbic acid (**E**,**F**) of loquat cvs. Sufaid and Surkh fruits kept at cold storage and 2-day shelf period. Different letters above bars indicate significant differences between treatments at *p* ≤ 0.05 (LSD test). Error bars represent ± SE; n = 36 (12 fruits × 3 replications).

TSS decreased progressively with advancing cold-storage interval when measured after 2 days at 20 °C ± 1 °C following each cold storage interval in both loquat cultivars (Sufaid and Surkh). In ‘Sufaid’, storage interval had a highly significant effect on TSS (*p* ≤ 0.01), and at 20 days of cold storage plus 2 days at 20 °C ± 1 °C, control fruit showed lower TSS (5.90 °Brix) than TG-treated fruit (7.22 °Brix) (Figure 4A). In ‘Surkh’, TSS was highly influenced by treatment, storage interval, and their interaction (*p* ≤ 0.01), with TSS remaining higher in TG-treated fruit than control at the final assessment point (7.33 vs. 6.21 °Brix) after 20 days of cold storage plus 2 days at 20 °C ± 1 °C (Figure 4B). TA declined markedly across storage intervals when assessed after 2 days at 20 °C ± 1 °C following each cold-storage interval in both cultivars. In ‘Sufaid’, TA differed significantly among treatments (*p* ≤ 0.05); by 20 days of cold storage plus 2 days at 20 °C ± 1 °C, TG-treated fruit retained higher TA (0.55%) than the control (0.30%) (Figure 4C). In ‘Surkh’, storage interval had a highly significant effect on TA (*p* ≤ 0.01), and TG-treated fruit consistently maintained higher TA than the control, reaching 0.50% versus 0.35% at 20 days of cold storage plus 2 days at 20 °C ± 1 °C (Figure 4D). Ascorbic acid content decreased with advancing storage interval when measured after 2 days at 20 °C ± 1 °C following each cold-storage interval in both loquat cultivars. In ‘Sufaid’, treatment, storage interval, and their interaction were non-significant (*p* ≥ 0.05), although TG-treated fruit showed numerically higher ascorbic acid than the control at the final assessment point (37.33 vs. 34.67 mg 100 g^−1^ FW) after 20 days of cold storage plus 2 days at 20 °C ± 1 °C (Figure 4E). In ‘Surkh’, treatment significantly affected ascorbic acid (*p* ≤ 0.05), with TG-treated fruit retaining substantially higher ascorbic acid than untreated fruit at 20 days of cold storage plus 2 days at 20 °C ± 1 °C (40.0 vs. 24.0 mg 100 g^−1^ FW) (Figure 4F).

### 3.4. Total Phenolic Content, Total Carotenoids and Antioxidant Activity

**Figure 5:**
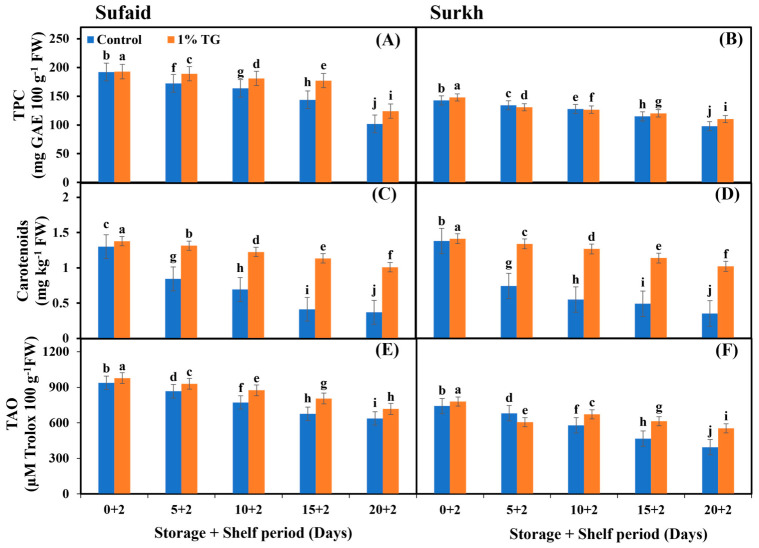
Effect of postharvest TG coating on changes in TPC (**A**,**B**), carotenoids (**C**,**D**) and TAO (**E**,**F**) of loquat cvs. Sufaid and Surkh fruits kept at cold storage and 2-day shelf period. Different letters above bars indicate significant differences between treatments at *p* ≤ 0.05 (LSD test). Error bars represent ± SE; n = 36 (12 fruits × 3 replications).

TPC declined progressively with advancing cold-storage interval when assessed after 2 days at 20 °C ± 1 °C following each interval in both loquat cultivars (Sufaid and Surkh). In ‘Sufaid’, TPC was highly influenced by treatment, storage interval, and their interaction (*p* ≤ 0.01), and TG-coated fruit consistently retained higher TPC than the control; at 20 days of cold storage plus 2 days at 20 °C ± 1 °C, TG-treated fruit maintained higher TPC (124.12 mg 100 g^−1^ FW) than non-treated fruit (101.79 mg 100 g^−1^ FW) (Figure 5A). In ‘Surkh’, both storage interval and treatment had highly significant effects on TPC (*p* ≤ 0.01), and TG-coated fruit showed better retention, reaching 110.01 mg 100 g^−1^ FW compared with 97.68 mg 100 g^−1^ FW in the control at 20 days of cold storage plus 2 days at 20 °C ± 1 °C (Figure 5B). Carotenoid content also decreased progressively across storage intervals when measured after 2 days at 20 °C ± 1 °C following each cold-storage interval in both cultivars. In ‘Sufaid’, treatment, storage interval, and their interaction had highly significant effects on carotenoids (*p* ≤ 0.01), and at 20 days of cold storage followed by 2 days at 20 °C ± 1 °C, TG-treated fruit retained higher carotenoids (1.01 mg 100 g^−1^ FW) than untreated fruit (0.37 mg 100 g^−1^ FW) (Figure 5C). In ‘Surkh’, carotenoids were likewise highly influenced by treatment, storage interval, and their interaction (*p* ≤ 0.01), with TG-treated fruit maintaining higher carotenoids than the control at the final assessment point (1.02 vs. 0.35 mg 100 g^−1^ FW) after 20 days of cold storage plus 2 days at 20 °C ± 1 °C (Figure 5D). Total antioxidant activity (TAO) declined with advancing storage interval when assessed after 2 days at 20 °C ± 1 °C following each cold-storage interval in both loquat cultivars. In ‘Sufaid’, storage interval, treatment, and their interaction significantly affected TAO (*p* ≤ 0.05), and at 20 days of cold storage plus 2 days at 20 °C ± 1 °C, TG-treated fruit maintained higher TAO (717.59 mg 100 g^−1^ FW) than untreated fruit (637.26 mg 100 g^−1^ FW) (Figure 5E). In ‘Surkh’, TAO was highly influenced by storage interval, treatment, and their interaction (*p* ≤ 0.01), and TG-treated fruit retained higher TAO than the control at the final assessment point, recording 531.38 mg 100 g^−1^ FW compared with 379.59 mg 100 g^−1^ FW after 20 days of cold storage plus 2 days at 20 °C ± 1 °C (Figure 5F).

### 3.5. Malondialdehyde Content, Hydrogen Peroxide Content and Electrolyte Leakage

**Figure 6:**
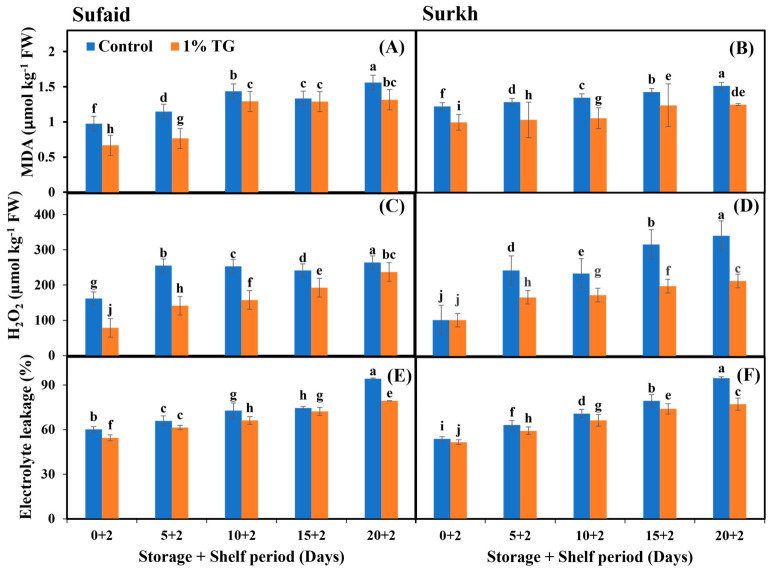
Effect of postharvest TG coating on changes in MDA (**A**,**B**), H_2_O_2_ (**C**,**D**) and electrolyte leakage (**E**,**F**) of loquat cvs. Sufaid and Surkh fruits kept at cold storage and 2 days shelf period. Different letters above bars indicate significant differences between treatments at *p* ≤ 0.05 (LSD test). Error bars represent ± SE; n = 36 (12 fruits × 3 replications).

MDA content increased with advancing cold-storage interval when assessed after 2 days at 20 °C ± 1 °C following each interval in both loquat cultivars (Sufaid and Surkh). In ‘Sufaid’, the effects of treatment, storage interval, and their interaction were non-significant (*p* ≥ 0.05); however, TG-treated fruit consistently showed lower MDA at the final assessment point (1.32 nmol 100 g^−1^ FW) than the control (1.56 nmol 100 g^−1^ FW) after 20 days of cold storage plus 2 days at 20 °C ± 1 °C (Figure 6A). In ‘Surkh’, storage interval, treatment, and their interaction were also non-significant (*p* ≥ 0.05), yet TG-coated fruit maintained lower MDA than the control at 20 days of cold storage plus 2 days at 20 °C ± 1 °C (1.25 vs. 1.52 nmol 100 g^−1^ FW) (Figure 6B), indicating a consistent numerical reduction in lipid peroxidation with TG application despite the lack of statistical significance. Hydrogen peroxide (H_2_O_2_) content increased across storage intervals when measured after 2 days at 20 °C ± 1 °C following each cold-storage interval in both cultivars. In ‘Sufaid’, H_2_O_2_ changed significantly across storage intervals (*p* ≤ 0.01), and TG application reduced oxidative accumulation at the final assessment point, with lower H_2_O_2_ in TG-treated fruit (237.14 µmol 100 g^−1^ FW) than in the control (264.04 µmol 100 g^−1^ FW) after 20 days of cold storage plus 2 days at 20 °C ± 1 °C (Figure 6C). In ‘Surkh’, storage interval, treatment, and their interaction were highly significant (*p* ≤ 0.01), and TG-treated fruit maintained substantially lower H_2_O_2_ than the control at 20 days of cold storage plus 2 days at 20 °C ± 1 °C (211.48 vs. 339.23 µmol 100 g^−1^ FW) (Figure 6D). Electrolyte leakage increased progressively with advancing cold-storage interval when assessed after 2 days at 20 °C ± 1 °C following each interval in both loquat cultivars. In ‘Sufaid’, storage interval had a highly significant effect (*p* ≤ 0.01), and TG reduced leakage at the final assessment point, with 79.49% in TG-treated fruit compared with 94.28% in the control after 20 days of cold storage plus 2 days at 20 °C ± 1 °C (Figure 6E). In ‘Surkh’, storage interval and treatment significantly influenced electrolyte leakage (*p* ≤ 0.05), and TG-treated fruit consistently maintained lower leakage, reaching 84.37% versus 94.84% in the control at 20 days of cold storage plus 2 days at 20 °C ± 1 °C (Figure 6F).

### 3.6. Enzymatic Analysis (SOD, POD, CAT and APX)

**Figure 7:**
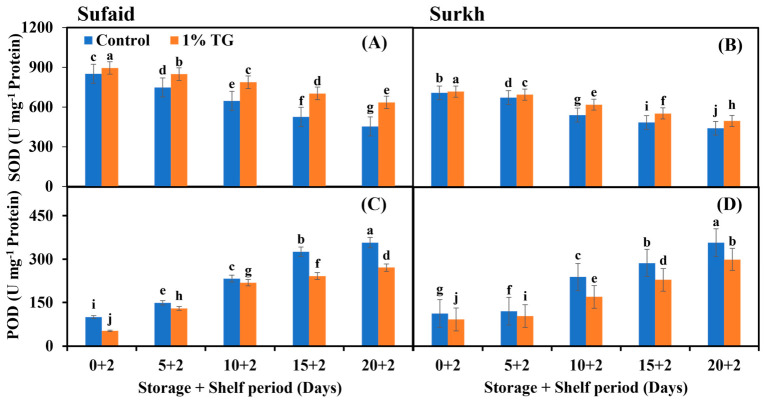
Effect of postharvest TG coating on changes in SOD (**A**,**B**) and POD (**C**,**D**) of loquat cvs. Sufaid and Surkh fruits kept at cold storage and 2-day shelf period. Different letters above bars indicate significant differences between treatments at *p* ≤ 0.05 (LSD test). Error bars represent ± SE; n = 36 (12 fruits × 3 replications).

SOD activity decreased with advancing cold-storage interval when assessed after 2 days at 20 °C ± 1 °C following each interval in both loquat cultivars (Sufaid and Surkh). In ‘Sufaid’, storage interval, treatment, and their interaction had highly significant effects on SOD (*p* ≤ 0.01), and TG-treated fruit retained higher SOD activity at the final assessment point, reaching 634.86 U mg^−1^ protein compared with 453.71 U mg^−1^ protein in the control after 20 days of cold storage plus 2 days at 20 °C ± 1 °C (Figure 7A). In ‘Surkh’, storage interval, treatment, and their interaction significantly influenced SOD activity (*p* ≤ 0.05), and TG-treated fruit maintained higher activity than the control at 20 days of cold storage plus 2 days at 20 °C ± 1 °C (499.59 vs. 443.64 U mg^−1^ protein) (Figure 7B). POD activity increased across storage intervals when measured after 2 days at 20 °C ± 1 °C following each cold-storage interval in both cultivars. In ‘Sufaid’, treatment, storage interval, and their interaction had highly significant effects on POD (*p* ≤ 0.01), and POD activity was markedly higher in control than in the TG-treated fruit at 20 days of cold storage plus 2 days at 20 °C ± 1 °C (357.15 vs. 270.30 U mg^−1^ protein) (Figure 7C). In ‘Surkh’, storage interval, treatment, and their interaction were also highly significant (*p* ≤ 0.01), and TG-treated fruit retained lower POD activity than untreated fruit at the final assessment point (298.78 vs. 357.11 U mg^−1^ protein) after 20 days of cold storage plus 2 days at 20 °C ± 1 °C (Figure 7D).

**Figure 8:**
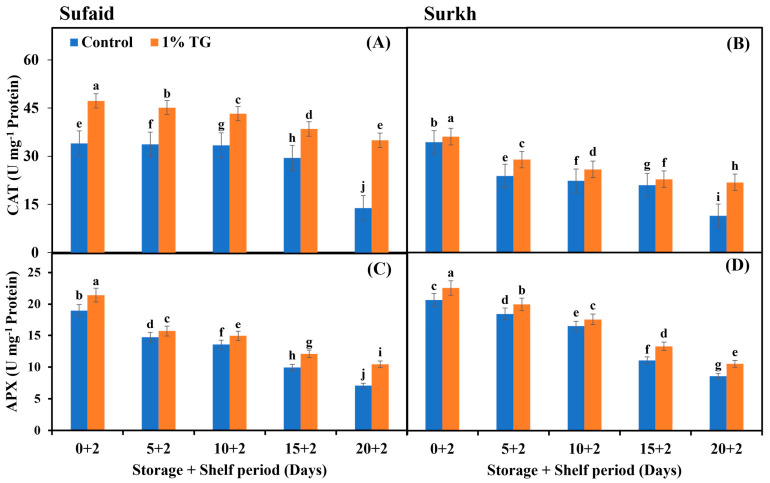
Effect of postharvest TG coating on changes in CAT (**A**,**B**) and APX (**C**,**D**) of loquat cvs. Sufaid and Surkh fruits kept at cold storage and 2-day shelf period. Different letters above bars indicate significant differences between treatments at *p* ≤ 0.05 (LSD test). Error bars represent ± SE. of means; n = 36 (12 fruits × 3 replications).

CAT activity varied significantly across storage intervals in both cultivars when assessed after 2 days at 20 °C ± 1 °C following each cold-storage interval, with TG consistently enhancing CAT activity relative to the control. In ‘Sufaid’, treatment and storage interval had highly significant effects on CAT (*p* ≤ 0.01), and TG-treated fruit maintained substantially higher CAT activity than the control after 20 days of cold storage plus 2 days at 20 °C ± 1 °C (34.98 vs. 13.88 U mg^−1^ protein) (Figure 8A). In ‘Surkh’, storage interval had a highly significant effect on CAT (*p* ≤ 0.01), and TG-treated fruit again retained higher CAT activity at the final assessment point (21.80 U mg^−1^ protein) than untreated fruit (11.39 U mg^−1^ protein) after 20 days of cold storage plus 2 days at 20 °C ± 1 °C (Figure 8B). APX activity generally decreased with advancing storage interval when measured after 2 days at 20 °C ± 1 °C following each cold-storage interval in both loquat cultivars, with TG tending to maintain higher APX than the control. In ‘Sufaid’, treatment, storage interval, and their interaction were highly significant (*p* ≤ 0.01), and TG-treated fruit exhibited higher APX at the final assessment point (10.43 U mg^−1^ protein) than the control (7.09 U mg^−1^ protein) after 20 days of cold storage plus 2 days at 20 °C ± 1 °C (Figure 8C). In ‘Surkh’, storage interval and treatment significantly affected APX (*p* ≤ 0.05), while their interaction was non-significant; TG-treated fruit retained higher APX than untreated fruit at 20 days of cold storage plus 2 days at 20 °C ± 1 °C (10.46 vs. 8.54 U mg^−1^ protein) (Figure 8D).

### 3.7. Sensory Attributes

**Figure 9:**
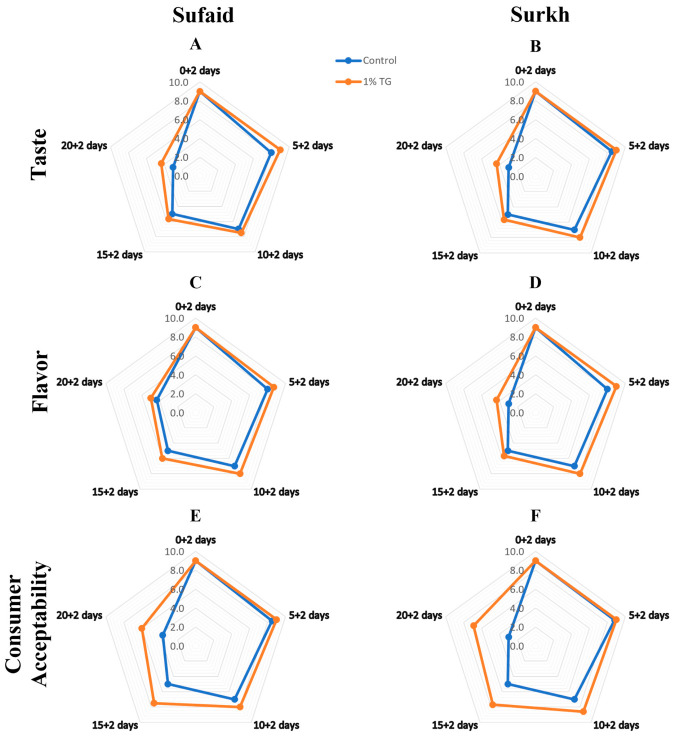
Effect of postharvest TG coating on changes in taste (**A**,**B**), flavor (**C**,**D**), and consumer acceptability (**E**,**F**) of loquat cvs. Sufaid and Surkh fruits kept at cold storage and 2-day shelf period; n = 36 (12 fruits × 3 replications).

Fruit taste declined progressively in both loquat cultivars (Sufaid and Surkh) during cold storage followed by 2 days of shelf period; however, 1% TG-treated fruits maintained higher taste scores and showed significant results than untreated controls. In ‘Sufaid’, TG-treated fruits exhibited a higher taste score (4.3) compared with control fruits (3.0) after 20 days of storage + 2 days shelf period (Figure 9A). A similar trend was observed in ‘Surkh’, where 1% TG-treated fruits retained a higher taste score (4.3) than untreated fruits (3.0) at the final evaluation point (Figure 9B). Fruit flavor decreased continuously during storage and shelf period in both cultivars, while TG coating effectively delayed sensory deterioration. After 20 days of storage + 2 days shelf period, ‘Sufaid’ fruits treated with 1% TG showed a higher flavor score (5.0) than control fruits (4.3) (Figure 9C). Likewise, in ‘Surkh’, coated fruits maintained a higher flavor score (4.3) compared with untreated control fruits (3.0) at the end of storage (Figure 9D). Consumer acceptability declined throughout storage in both loquat cultivars; however, TG-treated fruits consistently showed superior consumer acceptability compared with untreated fruits. In ‘Sufaid’, 1% TG-treated fruits recorded a higher acceptability score (6.0) than control fruits (3.7) after 20 days of storage + 2-day shelf period (Figure 9E). Similarly, in ‘Surkh’, TG-treated fruits maintained the highest acceptability score (6.9), whereas untreated fruits showed the lowest score (3.0) at the final storage interval (Figure 9F). Overall, these findings suggested that the postharvest application of 1% TG coating in cold storage and shelf period effectively helped preserve sensory quality attributes of loquat fruit, thus improving the acceptability and marketability of the fruit.

### 3.8. Correlation Analysis

**Figure 10:**
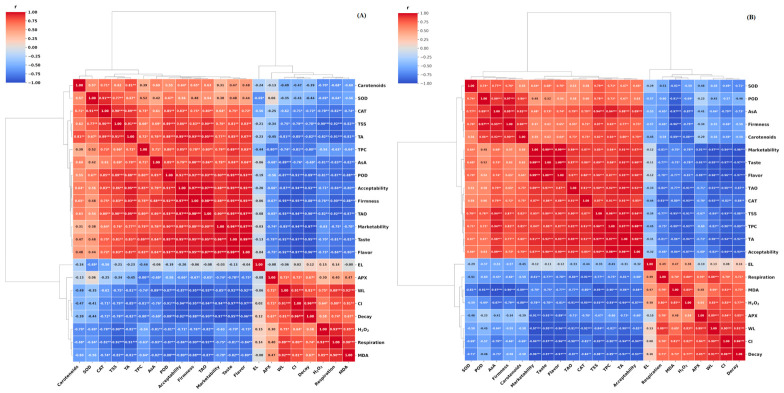
Clustered Pearson’s correlation coefficients among different quality, physiological, biochemical, sensory and antioxidant attributes of loquat fruit cultivars during post-storage ambient evaluation. Correlation analysis for cv. ‘Sufaid’ (**A**) and cv. ‘Surkh’ (**B**) following cold storage and subsequent assessment at 20 °C ± 1 °C. Red and blue colors represent positive and negative correlations, respectively, while dendrograms indicate hierarchical clustering of related variables. Statistical significance of correlations is indicated by *, **, and ***, representing significance levels of *p* ≤ 0.05, *p* ≤ 0.01, and *p* ≤ 0.001, respectively. Descriptions of all abbreviated terms are provided in the Materials and Methods section.

The fruit quality and the antioxidant-related traits recorded a strong positive correlation with each other in both cultivars and included TSS, TA, AsA, TPC, TAO, CAT, POD, taste, flavor, acceptability and marketability. These parameters were grouped closely together suggesting that they are coordinately regulated, in relation to fruit quality maintenance and antioxidant defense systems during storage. In contrast, the parameters related to stress and deterioration such as WL, CI, decay incidence, respiration rate, H_2_O_2_, and MDA showed very high inter-correlations and significant negative correlations with the quality and antioxidant parameters, respectively. The improvement in oxidative stress and physiological deterioration was accompanied by decrease in antioxidant capacity and sensory quality which corresponds to this pattern. The antioxidant enzymes (CAT, POD, SOD) and bioactive compounds (AsA, TPC, TAO) had strong positive correlation with sensory attributes and fruit quality parameters for the Sufaid cultivar (Figure 10A), while the oxidative damage markers (H_2_O_2_, MDA) were negatively correlated. In the Surkh cultivar (Figure 10B), quality traits also showed high positive correlations among each other, though the pattern of clustering suggested that the relationships between the antioxidant-related traits and sensory traits were stronger than the relationships between the deterioration-related traits. In Surkh, the negative correlations among oxidative stress and fruit quality parameters were also more significant. In general, the correlation analysis showed that there are significant differences between the antioxidant defense functions that promote fruit quality and the degradation processes that occur during storage, suggesting a key importance for antioxidant defense in maintaining postharvest fruit quality.

## 4. Discussion

Loquat fruit is sensitive to oxidative stress, membrane damage and accelerated senescence during the postharvest storage, and chilling injury with associated quality deterioration remains a major concern. Edible coatings could alleviate these disorders by controlling gas exchange, oxidative metabolism and cell defense mechanisms as semi-permeable barriers. Recent studies have confirmed that edible coatings effectively reduce chilling injury and oxidative stress in climacteric and non-climacteric fruits by modulating ROS metabolism and membrane stability during storage [14]. In this study, TG coating improved loquat storability through interconnected physiological and biochemical mechanisms rather than isolated effects on individual quality parameters. The semi-permeable TG layer likely moderated moisture loss and internal gas exchange, thereby slowing dehydration, respiration, and senescence metabolism. TG film formation is attributed to its polysaccharide-based hydrocolloid matrix, which forms a continuous viscoelastic barrier with selective permeability to O_2_, CO_2_, and water vapor, thereby regulating internal atmosphere and slowing metabolic transitions [39]. Recent studies on polysaccharide-based coatings [40,41] have highlighted their role in forming semi-permeable films that regulate gas exchange, reduce respiration rate, and delay ripening in fruit systems. Similar multifunctional preservation mechanisms have been widely reported for edible coatings, where restricted water diffusion and modified O_2_/CO_2_ exchange reduce metabolic activity and delay ripening progression [13]. Consequently, reduced weight loss, delayed softening, and lower respiration observed in TG-treated fruit appear to represent linked outcomes of slowed metabolic progression and improved cellular homeostasis. The reduced water loss under TG coating is particularly important because dehydration, firmness decline, respiration, and ripening progression are physiologically interdependent processes during loquat storage. Weight loss caused by vapor-pressure gradients commonly accelerates shrinkage, softening, and quality deterioration [6,42]. Similar reductions in weight loss and improved firmness retention have been reported in chitosan-coated mango [43], alginate-coated apple [44], and gum-based coated mango fruit during cold storage [45]. The lower weight loss observed in TG-coated fruit agrees with the protective barrier function of natural gum coatings against water-vapor diffusion [46]. This barrier effect also reduces cuticular transpiration and stabilizes epidermal cell turgor pressure, thereby maintaining surface integrity and delaying senescence-associated structural collapse [47]. By limiting dehydration and reducing metabolic activity, TG likely delayed cell-wall disassembly, pectin solubilization, and senescence-associated tissue weakening, explaining the superior firmness retention observed here, consistent with previous findings in TG-treated mango and apricot fruit [16,18]. This effect involves suppression of cell wall-degrading enzymes such as PME, PG, and cellulase, thereby maintaining middle lamella integrity and structural rigidity of parenchyma tissues. Simultaneously, suppression of respiration under TG coating likely moderates ripening-associated biochemical changes because respiratory metabolism drives substrate utilization, organic acid depletion, and accelerated senescence [48,49]. Reduced respiration may also preserve mitochondrial integrity and limit electron leakage, thereby reducing ROS generation at the organelle level.

These coordinated effects were reflected in the behavior of soluble solids, titratable acidity, and ascorbic acid. Lower TSS accumulation together with partial retention of TA suggests delayed ripening and slower utilization of respiratory substrates under TG treatment. Recent reports have shown that edible coatings effectively delay TSS accumulation and preserve TA and AsA by reducing metabolic respiration and oxidative degradation in sweet orange [50], bananas [51] and blueberries [52]. Because sugars and organic acids collectively define sweetness–acidity balance, preservation of these parameters may contribute to maintaining desirable taste, flavor perception, and sensory quality of litchi [53] and loquat fruit during storage [54]. Similarly, improved retention of ascorbic acid indicates reduced oxidative degradation and slower senescence progression. Beyond its nutritional role, ascorbic acid participates directly in ROS detoxification through the AsA–GSH cycle [55]; therefore, its preservation reinforces antioxidant defense and freshness-related quality. TG coating may further stabilize AsA recycling by reducing oxidative burden and sustaining redox buffering capacity during storage. The beneficial effects of TG coating on physicochemical quality were closely associated with enhanced tolerance to cold-storage stress. Chilling injury in loquat is strongly linked with membrane destabilization, oxidative burst, and loss of cellular compartmentation [56]. In the present study, reduced chilling injury, lower decay, and improved marketability appear to arise from common protective mechanisms involving reduced dehydration, moderated respiration, improved oxidative balance, and better membrane preservation. Because chilling injury often predisposes tissues to pathogen invasion, the suppression of decay under TG treatment is consistent with delayed senescence, improved firmness retention, and reduced tissue disruption. Several recent studies in chilling-sensitive fruits persimmon [57], guava [58] and strawberry [59] have demonstrated that membrane stabilization and reduced ROS accumulation are key mechanisms for mitigating chilling injury during cold storage. These improvements also have practical implications for consumer perception because appearance, texture, and physiological integrity strongly determine marketability and purchasing acceptance. Additionally, TG coating likely acts as a physical microbial barrier, limiting pathogen colonization and reducing infection sites on bell pepper surfaces [60].

A major component of TG-mediated protection appears to involve maintenance of antioxidant metabolism and cellular redox balance. Enhanced antioxidant enzyme activity under edible coating treatments has been consistently reported in bananas [61], grapefruit [62] and mango fruit [63] confirming activation of ROS-scavenging systems as a primary defense response during cold storage stress. The higher retention of phenolics, carotenoids, antioxidant activity, and ascorbic acid in TG-treated fruit collectively suggests preservation of antioxidant pools and reduced oxidative deterioration. Since phenolics and carotenoids contribute not only to antioxidant defense but also to fruit color, taste, visual appeal, and sensory characteristics, their preservation likely supported overall fruit quality and consumer acceptability [64]. Consistent with this interpretation, lower H_2_O_2_ accumulation and reduced MDA formation indicate improved ROS regulation and diminished lipid peroxidation under TG treatment. This suggests stabilization of membrane phospholipids and preservation of unsaturated fatty acids, thereby maintaining membrane fluidity under cold stress conditions [65]. Rather than representing separate biochemical observations, antioxidant metabolites, ROS accumulation, lipid oxidation, and membrane stability collectively describe a coordinated oxidative-stress response associated with delayed deterioration. This interpretation is further supported by electrolyte leakage and antioxidant enzyme responses in banana fruit [66]. Reduced electrolyte leakage confirms improved membrane integrity, which mechanistically links lower oxidative damage with reduced chilling injury, decay, and delayed senescence [67]. The coordinated behavior of antioxidant enzymes (SOD, CAT, APX, and POD) reinforces this interpretation. Enhanced enzyme activities under TG treatment suggest strengthening of the antioxidant defense network, allowing efficient ROS detoxification, reduced H_2_O_2_ accumulation, preservation of membrane functionality, and mitigation of cold-storage damage [68]. This enzymatic activation is linked to stress-responsive signaling pathways and transcriptional regulation of antioxidant genes under TG-induced mild abiotic stress modulation [69]. Therefore, antioxidant metabolites, enzymatic defenses, membrane preservation, and reduced oxidative injury should be considered interconnected protective responses rather than independent outcomes. These findings are consistent with recent literature demonstrating that polysaccharide-based edible coatings effectively integrate physical barrier effects with biochemical regulation of oxidative stress to delay postharvest deterioration in mango [70], apples [71], tomatoes [72] and black mulberry [73]. Overall, TG coating alleviated chilling-induced quality loss by reinforcing antioxidant defense systems and limiting ROS-mediated membrane damage, thereby delaying postharvest deterioration.

## 5. Conclusions

Postharvest application of 1% TG effectively improved storability and maintained eating quality of both loquat cultivars during 20 days of cold storage followed by 2 days of shelf-life at 20 °C ± 1 °C. Overall, TG treatment enhanced quality preservation by reducing WL, decay incidence, improving MI and sensory quality compared with untreated control fruits. TG application maintained key quality attributes, including fruit firmness, TSS, TA, and AsA, while enhancing bioactive compounds such as TPC, carotenoids, and TAO. It also improved membrane stability by reducing EL and accumulation of H_2_O_2_ and MDA, associated with enhanced antioxidant enzyme activities (SOD, CAT, and APX), thereby contributing to better fruit quality retention during storage and shelf-life. Correlation analysis further confirmed significant associations between enhanced antioxidant defense, improved membrane stability, and reduced oxidative stress. Together, 1% TG coating is a promising, low-cost, and easily applicable strategy for reducing postharvest losses and maintaining fruit quality under cold-chain storage and retail conditions. It can be readily adopted as a dip treatment in packinghouses due to its biodegradability and scalability. Future studies should further elucidate the molecular mechanisms underlying TG-mediated effects, particularly its regulation of antioxidant signaling, stress-responsive genes, and membrane stability pathways.

## Data Availability

The original contributions presented in this study are included in the article. Further inquiries can be directed to the corresponding authors.

## Abbreviations

The following abbreviations are used in this manuscript:

TG	Tragacanth Gum
AsA	Ascorbic Acid
CI	Chilling Injury
ROS	Reactive Oxygen Species
SOD	Superoxide Dismutase
CAT	Catalase
APX	Ascorbate Peroxidase
N	Newton
TSS	Total Soluble Solids
TA	Titratable Acidity
MI	Marketability Index
EL	Electrolyte Leakage
WL	Weight Loss
FW	Fresh Weight
TPC	Total Phenolic Content
TAO	Total Antioxidant Activity
MDA	Malondialdehyde
H_2_O_2_	Hydrogen Peroxide
POD	Peroxidase
CRD	Completely Randomized Design
